# Dietary Composition, Angiographic Coronary Disease, and Cardiovascular Outcomes in the WISE Study (Women’s Ischemia Syndrome Evaluation)

**DOI:** 10.3390/jcm12247762

**Published:** 2023-12-18

**Authors:** Brandon H. Schwartz, So Yung Choi, Anne Mathews, Monica Aggarwal, Eileen M. Handberg, Carl J. Pepine, William Rogers, Steven Reis, Galen Cook-Wiens, C. Noel Bairey Merz, Janet Wei

**Affiliations:** 1 Barbra Streisand Women’s Heart Center, Cedars-Sinai Smidt Heart Institute, Los Angeles, CA 90048, USA; b.h.schwartz92@gmail.com (B.H.S.); noel.baireymerz@cshs.org (C.N.B.M.); 2 Biostatistics and Bioinformatics Research Center, Cedars-Sinai Medical Center, Los Angeles, CA 90048, USA; soyung.choi@cshs.org (S.Y.C.); galen.cook-weins@cshs.org (G.C.-W.); 3 Division of Cardiovascular Medicine, University of Florida College of Medicine, Gainesville, FL 32611, USA; anne.mathews@ufl.edu (A.M.); monica.aggarwal@medicine.ufl.edu (M.A.); eileen.handberg@medicine.ufl.edu (E.M.H.);; 4 Division of Cardiovascular Disease, University of Alabama at Birmingham, Birmingham, AL 35294, USA; wrogers@uab.edu; 5 Division of Health Sciences, University of Pittsburgh, Pittsburgh, PA 15260, USA; sreis@pitt.edu

**Keywords:** chest pain, acute coronary syndrome, ischemia, women, mortality, coronary artery disease, diet, INOCA

## Abstract

Background: Studies relating diet to angiographic coronary artery disease (CAD) and subsequent major adverse cardiac events (MACE) in women are limited. Information on diet was collected in the Women’s Ischemia Syndrome Evaluation (WISE), a prospective cohort study of symptomatic women referred for coronary angiography to evaluate suspected ischemic heart disease. Methods: A consecutive subgroup (*n* = 201 of 936) of enrolled women completed the modified Block food frequency questionnaire (FFQ). Data on outcomes were collected and adjudicated after 8-year follow-up. A set of logistic regression models were fitted for non-obstructive versus obstructive coronary stenosis (<50% versus ≥50%). Cox proportional hazard regression models were fitted for outcomes, with each dietary composition variable adjusted for the degree of coronary stenosis. Results: At baseline, the subgroup cohort was 58 ± 12 years old with a body mass index (BMI) of 30 ± 7 kg/m^2^. An increased proportion of calories consumed from protein was associated with higher levels of baseline obstructive coronary stenosis. Those individuals who ate a higher amount of protein, carotene, and servings of vegetables and meat, however, were each associated with lower subsequent adverse outcomes, respectively. Conclusions: Among women undergoing coronary angiography for suspected CAD, a higher percentage of protein intake was associated with higher baseline stenosis severity; however, the amount of protein intake, vegetable, meat, and carotene intake, was conversely associated with subsequent lower adverse cardiovascular outcome risk.

## 1. Introduction

The medical community continues to search for the holy grail in terms of optimal diet that will improve cardiovascular outcomes. However, nutrition data are fraught with inaccuracies and assumptions. There are many types of research designs within nutrition, ranging from costly feeding trials to less reliable observational studies [1]. Food preparation and selection can also vary widely among individuals. People are also quite different in terms of food choices, metabolism, and recall for food questionnaires. They may feel guilt or shame about their decisions and thus misreport data. Furthermore, nutrition databases and the breakdown of macronutrients are hard to quantify and extrapolate for an individual’s consumption. Despite these inherent challenges with obtaining and interpreting nutrition data, it is important to evaluate the relationship between nutrition and health outcomes to generate hypotheses for clinical trials.

Cardiovascular disease is the leading killer of adults worldwide, yet there is a paucity of data concurrently interrogating relationships between dietary composition, coronary stenosis, and subsequent cardiovascular outcomes, including mortality, particularly in women. Prior studies have suggested often contradictory data with respect to dietary composition analysis and outcomes. A study assessing dietary risk factors for obstructive coronary disease with food frequency questionnaires (FFQs) found that protein intake predicted the presence of obstructive CAD for those undergoing invasive angiography in a mixed-sex cohort [2]. This was further verified by mechanistic animal data showing that high protein diets increase atherosclerotic plaque formation in male mice via the mammalian target of rapamycin (mTOR) pathway [3]. Another study in a mixed cohort of men and women showed that increased consumption of red meat, eggs, and chicken was positively correlated with angiographic obstructive coronary artery disease (CAD), defined as >50% stenosis [4]. Other prospective cohort studies have evaluated dietary composition in relation to CAD outcomes, specifically in women. Among the largest, the Nurses’ Health Study enrolled over 100,000 female nurses who completed food recall questionnaires. Several analyses of the primary data provided evidence that a diet rich in fruits and vegetables was associated with a reduced risk of CAD [5]. Additionally, the Iowa Women’s Health Study observed that higher consumption of protein from red meat was associated with subsequent increased death from all-cause mortality [6].

However, the level of coronary stenosis or cardiovascular outcomes, including death, have not been reported in longitudinal studies concurrently evaluating both outcomes in a single cohort of women. The disproportionate representation of ischemic heart disease (IHD), such as ischemia with no obstructive CAD (INOCA), heart failure with preserved ejection fraction (HFpEF), stroke, osteoporosis, and dementia in women compared to men that contribute to all-cause mortality suggests that the investigation of sex-specific nutrition related to longer-term aging is needed [7]. Despite the 2021 American Heart Association (AHA) guidelines for cardiovascular health recommending eating a diet rich in fruits and vegetables, focusing on plant proteins, choosing whole grains over refined grains, and limiting saturated fat content, they do not specify diets based on age groups or sex [8]. Accordingly, we sought to assess associations between dietary composition, baseline coronary angiography findings, and subsequent outcomes, including mortality in our cohort of women undergoing evaluation for suspected IHD.

## 2. Methods

The Women’s Ischemia Syndrome Evaluation (WISE, NCT 00000554) is a four-center prospective cohort study designed to explore myocardial ischemia in women and optimize the evaluation of symptoms and diagnostic testing for IHD [9]. The total enrollment included 936 women who underwent clinically indicated coronary angiography as part of routine care for signs and symptoms of IHD between 1996 and 2000. Incident major adverse cardiovascular events (MACE), such as all-cause mortality, myocardial infarction (MI), or stroke, were adjudicated after an average of 8-year follow-up. 

As part of the study, a subset of 223 consecutive women completed a modified Block FFQ based on the Study of Women’s Health Across the Nation (SWAN) [10]. The FFQ used was based on the Dietary Guidelines for Americans of the time and the guidance system for the US public, the Food Guide Pyramid [11]. The foods selected for inclusion on the FFQ were based on the National Health and Nutrition Exam Survey (NHANES) III dietary recall data, which has been validated in several adult populations [12]. Respondents reported their dietary intake of >100 food items over the past year with categories ranging from “never” to “twice per day”, including estimated portion size. Overall, 201 (90%) women provided complete data with the FFQ and were included in this sub-analysis. Data from the retrospective 1-year food recall included estimated daily intake of macronutrients (grams), percent of calories from fat, protein, carbohydrates, and sweets, and food group servings. Food group servings were (1) fats (fats, oils, and sweets), (2) milk (milk, yogurt, and cheese), (3) meat (meat, poultry, fish, dry beans, eggs, and nuts), (4) vegetables, (5) fruits, and (6) bread (bread, cereal, rice, and pasta). Other nutrient components, including carotene (per 100 ug) and cholesterol (grams), were included to help differentiate protein consumption into plant-based and meat-based. Completed FFQs were analyzed using NutritionQuest, and dietary output was returned to researchers.

All coronary angiograms were analyzed quantitatively and qualitatively at the WISE angiographic core laboratory (Rhode Island Hospital, Providence, RI) by investigators masked to all other WISE data [13]. The segments were measured quantitatively on cine film using electronic calipers or a cine projector-based technique. The presence of obstructive CAD was defined as ≥50% diameter stenosis in ≥1 major epicardial coronary artery. Minimal CAD was defined as ≥20% but <50% stenosis, and no CAD as <20% stenosis. The majority of women (88%) underwent an angiogram prior to or on the same date as the FFQ, and the incidence of MACE and levels of stenosis were not different from those who filled out the questionnaire after their angiogram. At the time, those with obstructive CAD would have been generally recommended as per clinical care to consume a diet with plenty of fruits, vegetables, and whole grains, with a primary emphasis on reducing saturated fat and cholesterol intake [14].

Classification of deaths as cardiovascular versus non-cardiovascular was adjudicated by a committee masked to identify information obtained on the death certificates. The adjudication protocol specified that other etiologies, including strokes, venous thrombosis, pulmonary hemorrhage/bleeding vasculitis, a renal disease associated with hypertensive heart, and sudden death without any additional information, were classified as cardiovascular deaths. 

Characteristics of the women’s diet composition were summarized using mean with standard deviation (±SD) for continuous variables and frequency as a percentage for categorical variables and compared across different degrees of coronary stenosis (<20%, 20–49%, and >50%) using Kruskal–Wallis rank sum test, Pearson’s Chi-squared test, or Fisher’s exact test, as appropriate. A series of logistic regression models were fitted for the degree of coronary stenosis (<50% versus ≥50%), with each dietary variable adjusted for baseline risk factors (age, BMI, history of hypertension, diabetes, dyslipidemia, and smoking status), and medications (use of statins, aspirin, angiotensin-converting enzyme inhibitor (ACE-i), or angiotensin receptor blocker (ARB)). A series of Cox proportional hazard regression models were fitted for MACE (stroke and myocardial infarction) or death, adjusting for each dietary variable, stenosis levels, baseline risk factors, and medications. 

Plasma levels of interleukin 6 (IL-6) and tumor necrosis factor-alpha (TNF-a) were measured from plasma collected at study entry using a commercially available enzyme-linked immunosorbent assay kit. Levels of high-sensitivity CRP (hsCRP) and serum amyloid A (SAA) were measured by high-sensitivity. All samples were assayed at a core laboratory using previously validated techniques.

## 3. Results

The average age of the cohort was 58 years old, with a BMI of 30. The majority of participants had hypertension, hyperlipidemia, or non-obstructive CAD, defined as <50% stenosis. Baseline food frequency questionnaire results were compiled and stratified by percent stenosis. As a cohort, participants did not meet the recommendations for fruit and vegetable consumption and reported an average intake of 12% of calories from saturated fat and 209 mg of dietary cholesterol per day. 

Logistic regression analysis demonstrated the association of each dietary variable with the likelihood of obstructive stenosis (defined as >50%), adjusted for age, BMI, history of hypertension, diabetes, dyslipidemia, smoking status, use of statin, aspirin, and ACE-i/ARB (Table 1). A set of regression models were analyzed using the above baseline characteristics for each dietary variable. Increased consumption of protein, as a proportion of total calories, was significantly associated with obstructive stenosis in women when adjusted for risk factors and baseline medications.

A Cox proportional hazard regression analysis was performed to assess the relationship of each dietary variable to adjudicated MACE and all-cause death (Table 2), adjusted for % stenosis (<20%, 20–49%, or >50%), age, BMI, history of hypertension, diabetes, dyslipidemia, smoking status, use of statin, aspirin, and ACE-i/ARB. There were 32 documented adverse cardiovascular events among the women, including 16 deaths, 8 MIs, and 8 strokes. The amount of protein consumption in grams, carotene (per 100 ug), servings of vegetables, and servings of foods from the meat group were each independently associated with lower MACE risk. 

Additional analysis of the above data incorporating baseline inflammatory markers (C-reactive protein, IL-6, SAA, and TNF-a) showed no significant difference in the main results or outcomes.

## 4. Discussion

In our prospective cohort of women with suspected myocardial ischemia undergoing coronary angiography and responding to a validated dietary composition measurement, higher protein consumption as measured using the percent of daily caloric intake was associated with a higher degree of coronary stenosis after adjusting for cardiovascular risk factors. On the other hand, the amount of protein and carotene consumed, as well as servings of vegetables and food from the meat group, were individually associated with lower MACE risk. This is the first known cohort study of women evaluating concurrently the associations between dietary composition, specifically macronutrients, and both baseline coronary stenosis and cardiovascular outcomes, including all-cause mortality in women. 

While a higher percentage of protein consumption of total calories was associated with higher levels of obstructive CAD, the absolute amount of protein consumption was associated with subsequent better cardiovascular outcomes in our cohort of women. Unfortunately, we are unable to determine the breakdown of protein into plant-based versus animal products in our cohort based on the FFQ. However, the inclusion of carotene (as a surrogate for plant consumption) and cholesterol (as a surrogate for meat consumption) helps approximate the distribution. A meta-analysis by Naghshi et al. showed a significant inverse relationship between protein intake and all-cause mortality in 31 cohort studies, including both men and women [15]. These investigators concluded that women who consumed higher amounts of total protein had lower all-cause mortality and that plant protein was associated with a lower risk of cardiovascular disease mortality at 15 years in both men and women. 

Our mortality results may be driven by a higher protein diet effect on reducing cardiometabolic risk, including improved blood pressure and decreased glycosylated hemoglobin levels and cholesterol, as described in the Pro-Heart trial [16]. Furthermore, malnutrition may play a part in cardiovascular risk and poor outcomes. In a cohort of men and women over 60 years old with IHD undergoing percutaneous coronary interventions (PCI), malnutrition, as defined by the Controlling Nutritional Status (CONUT) score, was directly associated with the risk of all-cause mortality [17]. Additionally, an analysis of the larger WISE cohort showed that 70% of the women studied had low functional capacity, and those with higher reported physical fitness scores were associated with less risk of MACE or CAD [18]. Thus, despite the majority of our population being overweight, they may also be more likely to be frail and unfit. It would make sense that higher protein consumption would counteract the deleterious effects of undernutrition, particularly in aging women. Another factor that may improve mortality in this cohort is less frailty. A subset of over 20,000 women aged 65–79 in the Women’s Health Initiative who consumed higher protein were found to have a lower risk of frailty [19]. 

Dietary recommendations have changed over the past 25 years since the initial enrollment of our cohort of women. The Food Guide Pyramid was released to assist Americans with healthy food choices, focusing on grain products, vegetables, and fruits while minimizing saturated fat, cholesterol, and sweets [20]. Other recommendations included balancing food intake with physical activity and minimizing alcohol use. While the basic tenets of food consumption have not changed much, the most recent AHA dietary guidelines more specifically break down food choices and healthy sources of protein, promoting the Mediterranean diet, Dietary Approaches to Stop Hypertension (DASH), style, and healthy vegetarian diets [8]. Additionally, they recognize inherent challenges to achieving and adhering to a heart-healthy diet when it comes to social determinants of health. The medical community needs to better encourage and promote the adoption of healthier eating habits and motivate behavioral change. 

## 5. Limitations

While our data are both cross-sectional and prospective, several limitations should be noted. Firstly, the FFQ does not detail the source of protein consumption. It would be useful to determine if protein from animal or plant sources was more protective against MACE and all-cause mortality. While we used cholesterol and carotene as surrogates for meat and vegetable consumption, it does not separate out dairy sources of protein or non-carotenoid vegetable protein. Secondly, there are no follow-up food questionnaires or angiograms to assess any dietary habits or angiographic status changes that could relate to the outcomes. Because the FFQ was generally assessed after the angiogram, there may be a bias toward reporting improved food outcomes or a significant dietary change after discovering CAD. Thirdly, the cohort is a small sample size and limited to a subgroup of WISE women and, therefore, may not represent the entire cohort or a direct comparison to similar cohorts of men. Finally, the statistically significant higher protein consumption measured using the percent of daily caloric intake across the stenosis groups was only a 1% difference, which may not be clinically meaningful.

## 6. Conclusions

Among women undergoing coronary angiography for suspected CAD, dietary composition of higher percent protein intake was associated with higher stenosis severity, but absolute protein intake, servings of vegetables and meat, and carotene intake were conversely associated with lower MACE. Further investigation is needed to better understand the role of dietary composition, CAD, and prognosis among women with IHD. 

## Figures and Tables

**Table 1:** Ordinal logistic regression of % stenosis.

	Stenosis (≥50% vs. <50%)		
Predictors	Odds Ratio	95% CI	*p*
Amounts			
Calories (per 100 Kcal)	0.98	0.90–1.06	0.694
Protein (per 10 g)	1.04	0.86–1.22	0.653
Total Fat (per 10 g)	0.95	0.80–1.10	0.547
Saturated Fat (per 10 g)	0.81	0.48–1.27	0.400
Carbohydrate (per 10 g)	0.99	0.93–1.05	0.797
Fiber (per 10 g)	1.61	0.60–4.13	0.328
Carotene (per 100 ug)	0.99	0.96–1.01	0.237
Cholesterol (per 10 mg)	0.98	0.94–1.01	0.210
**Servings**			
Vegetable Group	1.39	0.86–2.29	0.185
Fruit Group	1.02	0.64–1.62	0.937
Meat Group	1.13	0.62–1.80	0.656
Dairy Group	1.07	0.73–1.56	0.739
**Macronutrients and Sweets, as percent**			
Fat	0.98	0.94–1.02	0.358
Protein	1.14	1.03–1.27	**0.014**
Carbohydrate	1.00	0.97–1.04	0.981
Sweet	0.99	0.95–1.02	0.541

Adjusted for age, BMI, histories of hypertension, diabetes, dyslipidemia, smoking status (ever vs. never), uses of statins, aspirin, ACE/ARB, CRP, IL6, SAA, and TNFA.

**Table 2:** Cox proportion hazard regression model of death or MACE.

	Death or MACE Censored at Final Contact Date		
Predictors	Hazard Ratio	95% CI	*p*
Amounts			
Calories (per 100 Kcal)	0.92	0.82–1.03	0.134
Protein (per 10 g)	0.67	0.50–0.91	0.010
Total Fat (per 10 g)	0.84	0.67–1.04	0.108
Saturated Fat (per 10 g)	0.59	0.31–1.13	0.112
Carbohydrate (per 10 g)	0.96	0.89–1.04	0.310
Fiber (per 10 g)	0.61	0.19–1.94	0.401
Carotene (per 100 ug)	0.96	0.92–0.99	0.019
Cholesterol (per 10 mg)	0.98	0.94–1.01	0.210
**Servings**			
Vegetable Group	0.49	0.24–0.98	0.043
Fruit Group	1.01	0.61–1.65	0.979
Meat Group	0.32	0.14–0.75	0.009
Dairy Group	0.86	0.54–1.37	0.525
**Macronutrients and Sweets, as percent**			
Fat	0.96	0.92–1.01	0.103
Protein	0.93	0.84–1.03	0.181
Carbohydrate	1.02	0.23–1.06	0.285
Sweet	1.01	0.97–1.04	0.668

Adjusted for levels of stenosis (<20%, 20–49%, and ≥50% stenosis), age, BMI, histories of hypertension, diabetes, dyslipidemia, smoking status (ever vs. never), uses of statins, aspirin, ACE/ARB, CRP, IL6, SAA, and TNFA.

## Data Availability

The data presented in this study are available upon request.

## Abbreviations

MACE	major adverse cardiovascular events
mTOR	mammalian target of rapamycin
CAD	coronary artery disease
WISE	Women’s Ischemia Syndrome Evaluation
FFQ	food frequency questionnaire
BMI	body mass index
IHD	ischemic heart disease
INOCA	ischemia with no obstructive arteries
HFpEF	heart failure with preserved ejection fraction
AHA	American Heart Association
SD	standard deviation
MI	myocardial infarction
SWAN	Study of Women’s Health Across the Nation
PCI	percutaneous coronary intervention
CONUT	controlling nutrition status
DASH	Dietary Approaches to Stop Hypertension
ACE-I	angiotensin-converting enzyme inhibitor
ARB	angiotensin receptor blocker
NHANES	National Health and Nutrition Examination Survey
LDL	low-density lipoprotein
CRP	c-reactive protein
IL-6	interleukin-6
SAA	serum amyloid a
TNF-a	tumor necrosis factor-alpha
G	gram
Kcal	kilocalorie
